# Commentary: Active Preconditioning With Blood Flow Restriction or/and Systemic Hypoxic Exposure Does Not Improve Repeated Sprint Cycling Performance

**DOI:** 10.3389/fphys.2020.00122

**Published:** 2020-02-27

**Authors:** Hubert Bourgeois, Pénélope Paradis-Deschênes, François Billaut

**Affiliations:** Département de kinésiologie, Université Laval, Québec, QC, Canada

**Keywords:** blood-flow restriction, ischemic preconditioning, hypoxia, warm-up, performance

## Introduction

We read with great interest the study of Aebi et al. (2019) on the lively topic of hypoxic conditioning for sport performance. We congratulate the authors for their excellent work, which confirms, to some extent, some of our previous findings (Fortin and Billaut, 2019), and we also wish to comment on two points of importance: (i) the differences between ischemic preconditioning (IPC) and blood flow restricted exercise (BFR) as well as (ii) the importance of the practical nature of preconditioning protocols (i.e., warm-up) used in sport-oriented studies.

We believe that these two points are paramount for clarifying and, perhaps, unifying the scientific community with regards to the use of varied terms and modalities, ultimately for the purpose of providing clear, practical, and evidence-based recommendations to athletes and coaches.

## Intrinsic Differences Between IPC and BFR

The varied terminologies used in the scientific literature may be confusing for practitioners. We note that while Aebi and colleagues mentioned on several occasions the term “ischemic preconditioning” (IPC), they actually did not completely prevent the blood flow to the legs by using 60% of total occlusion pressure (equivalent to ~120 mmHg). In fact, they rather used a form of blood flow restriction during exercise to precondition physiological functions during a subsequent repeated-sprint exercise. Furthermore, the acronyms IPC and BFR are used almost interchangeably in some parts of the manuscript, whereas they relate to different modalities. We believe precision regarding terms and, therefore, the interpretation of the potency of the modalities is needed since both maneuvers are purposely employed to induce specific physiological responses and adaptations.

Typically, IPC is used at rest as a preconditioning tool *per se* and with very high pressure in order to occlude both the arterial and venous circulation. In fact, the attainment of occlusion is considered a determining factor for the effectiveness of the IPC-induced protective effects (Hughes et al., 2018). Performing cycles of occlusion/reperfusion can induce humoral, vascular and metabolic responses immediately (Paradis-Deschênes et al., 2016) and up to several days after (Jones et al., 2014) and is generally employed to acutely enhance physical capacity as well as for medical purposes (Tapuria et al., 2008; Salvador et al., 2016). On the other hand, BFR rather impedes the arterial inflow while blocking or impeding the limb venous outflow during exercise (Loenneke et al., 2014). Contrary to IPC, high pressure during exercise may impact the clinical utility of BFR and have a detrimental effect (Hughes et al., 2018). To date, BFR has typically been used to enhance training-induced peripheral adaptations (Christiansen et al., 2019). Hence, we certainly acknowledge the complexity of the experimental design (with separate and combined stresses) used by Aebi et al. and appreciate the difficulty of juggling with terms that do not quite qualify to introduce new concepts. To this end, we have proposed the so-called acronym “WFR” to define a blood flow-restricted situation specifically applied to warm-up for preconditioning purposes (Fortin and Billaut, 2019).

## Potentiating the Effects of a Warm-up in the Sport Context

The warm-up is a form of preconditioning which ergogenic effects are well-demonstrated. While extensive research has been devoted to improving the effects of a warm-up, the application of blood flow restriction (with its hemodynamic/hypoxic effects mimicking those of high-intensity exercise) is new and appealing. To our best knowledge, our laboratory reported the first data of the effects of a warm-up combined with BFR (Fortin and Billaut, 2019). Warming-up with blood flow restriction (WFR), albeit using elastic bands and a perceived pressure rather than a more expensive cuff system to try and maintain a constant pressure, enhanced muscle hemodynamics and oxygenation (derived from near-infrared spectroscopy) during twelve 20-m running sprints in trained American football players. Despite increases in blood volume and oxygenation during both sprint and recovery phases, performance was not meaningfully enhanced, which was confirmed by our colleagues using eight 10-s cycling sprints with trained cyclists and untrained healthy men. We noted, however, that running time in the last sprints repetitions tended to be faster compared to placebo in our study, indicating the potential of WFR for longer activities.

WFR may also provide a more practical way of ripping BFR benefits during a warm-up within a sport context. In fact, we reported on the feasibility of using blood-flow restriction within an actual competition-specific ~20-min warm-up routine (including individualized activation exercises, dynamic stretches, and sprints) in American football players without any issues. We believe this may be more easily implemented by athletes in competitive settings than the ~90-min sequence suggested by Aebi et al., including an easy warm-up, 5-min flow-restricted cycles during exercise, passive rest, and a re-warm-up before a given performance.

## Conclusion

In summary, we would like to highlight the mechanistic value of combining experimental conditions as used by Aebi et al. (2019), which may provide precise physiological responses to hypoxic vs. hyperemic preconditioning. Our commentary aims at highlighting the importance of clearly delineating modalities for the practitioner and for future research, and, from this perspective, WFR adds a specific, targeted, and feasible modality of preconditioning to the broader IPC and BFR literature. Taken together, these data suggest a potential benefit of hypoxic/hyperemic preconditioning for activities involving more bouts of maximal efforts or, perhaps, when exercising at altitude. Hypoxia and hyperemia represent stressors that human tissues readily respond to, and there is therefore scope for fundamental and applied research to use preconditioning in performance and health.

## Author Contributions

FB conceptualized the commentary. FB wrote the manuscript with revisions from HB and PP-D. All authors reviewed and agreed upon the final manuscript.

### Conflict of Interest

The authors declare that the research was conducted in the absence of any commercial or financial relationships that could be construed as a potential conflict of interest.
